# The adaptability of facultative parthenogenesis and ‘multiple embryos per eggcase’ as alternative reproductive strategies in Chondrichthyes

**DOI:** 10.1098/rsos.242030

**Published:** 2025-04-30

**Authors:** Joel Harrison Gayford

**Affiliations:** ^1^ James Cook University, Townsville, Australia; ^2^ Shark Measurements, London, UK

**Keywords:** evolution, fecundity, parthenogenesis, polyembryony, reproductive mode, sharks, elasmobranchs, oviparity, viviparity, mate choice

## Abstract

Chondrichthyans (sharks, rays and chimaera) are a fascinating case study through which to examine reproductive biology. While most vertebrate lineages have converged upon either placental viviparity or oviparity, chondrichthyans exhibit unparalleled diversity in reproductive mode and other aspects of reproductive biology. Despite this, our understanding of reproductive variation in this clade remains incomplete. Namely, several ‘unusual’ reproductive traits have been repeatedly observed in some chondrichthyan taxa, but we have little to no understanding of their adaptive value. Here, I focus on two traits (facultative parthenogenesis and the deposition of multiple embryos with separate yolks within a single eggcase (MEPE)), which theoretically result in exponential increases in fecundity. I discuss the theoretical fitness consequences of these traits, the range of species and eco-evolutionary contexts in which they have been documented and major open questions regarding their prevalence and evolutionary consequences. It appears likely that both facultative parthenogenesis and MEPE are adaptive in at least some chondrichthyan taxa and ecological contexts. However, additional data are needed to determine the true effect of these traits on lifetime fecundity, the frequency with which they occur, potential population-level effects and cues or triggers that might favour switches from ‘typical’ sexual reproduction to parthenogenesis or MEPE.

## 1. Introduction

Chondrichthyans (sharks, rays and chimaera) exhibit unparalleled reproductive variation relative to other vertebrates [1]. Oviparity, oophagy, histotrophy and placental viviparity (placentatrophy) have all evolved on several occasions across different vertebrate lineages [2–5], but in the vast majority of cases, reproductive diversity has been eroded over time to either oviparity or placental viviparity [6]. Chondrichthyans are unusual in this regard, as all of these aforementioned reproductive modes are observed in extant taxa [7], as well as additional modes such as yolk-only viviparity [6]. This remarkable reproductive diversity probably evolved from an initial ancestral state of oviparity, as observed in other vertebrate clades [4]. The evolution of matrotrophic modes such as placental viviparity appears to have profound consequences for the evolution of life-history traits and ecological lifestyle, as well as for diversification dynamics ([1,8–10]). However, there is still uncertainty regarding the evolutionary drivers of specific transitions in reproductive mode within Chondrichthyes [10].

Apparent reproductive and developmental abnormalities and peculiarities have long been documented in chondrichthyan taxa ([7,11,12] and references therein). Examples of unusual reproductive strategies that appear ubiquitous (and presumably adaptive) in the taxa in which they have been observed include intra-uterine cannibalism [13], whereas isolated cases of monozygotic or dizygotic twins in chondrichthyan species are generally regarded as true aberrations with no adaptive benefit [12,14]. As observational studies accumulate, however, some aspects of chondrichthyan reproductive biology previously assumed to be rare or unusual are increasingly thought to be commonplace. Examples include multiple paternity and female sperm storage, both of which were initially documented in few species [15,16] but now appear to be present across much of chondrichthyan diversity [17,18]. Studies directly addressing the adaptive significance of reproductive traits in Chondrichthyes are rare, in large part due to missing data from the literature. In light of this, I herein shall use the term ‘alternative reproductive strategy’ to refer to any reproductive trait that has been observed in some chondrichthyan taxa, but which does not appear to be ubiquitous and the adaptive basis of which remains uncertain.

Herein, I focus on two alternative reproductive strategies that despite being the focus of several recent studies remain poorly understood from an evolutionary perspective, at least within Chondrichthyes. The first of these strategies is facultative parthenogenesis, the occurrence of asexual reproduction (specifically, by development of an unfertilized egg cell) in species that typically reproduce sexually [19]. Subsequently, I will consider the apparently rarer case of multiple embryos (attached to distinct yolk sacs) being identified within a single eggcase [20]. In each case, I will consider the theoretical and empirical support for positive or negative individual fitness consequences stemming from the possession of the trait in question, before commenting on potential cues, triggers and population-level effects. Finally, I highlight remaining open questions and areas in which additional studies are required to improve our understanding of chondrichthyan reproductive biology and its evolutionary underpinnings.

## 2. Facultative parthenogenesis

**Figure 1.**
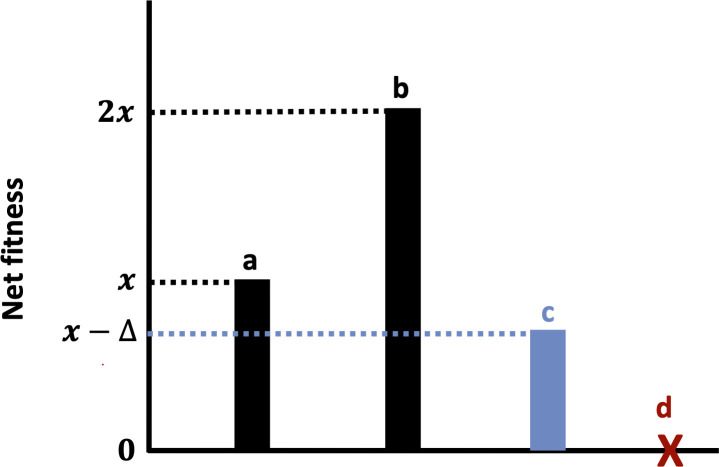
Theoretical lifetime net fitness values vary under different reproductive strategies and ecological contexts in a facultatively parthenogenetic species: sexual reproduction under ‘typical’ ecological conditions (a), asexual reproduction with no fitness costs (b), asexual reproduction with fitness costs (of magnitude x+∆) (c), sexual reproduction in the absence of potential mates (d). Note that this final scenario does not account for potential sperm storage.

Parthenogenesis is a specific form of asexual reproduction, most commonly (in vertebrates) occurring by fusion of an egg with a polar body (terminal fusion automixis), and resulting in the successful development of offspring without fertilization [21]. While obligate parthenogenesis is somewhat rare among vertebrates, facultative parthenogenesis is more abundant and appears to be present in representatives of most major vertebrate lineages besides mammals [21,22]. Parthenogenesis is theoretically favourable from the standpoint of individual fitness as it doubles the number of offspring produced in a single reproductive cycle relative to sexual reproduction (figure 1a,b; [23,24]). This observation has been termed the ‘twofold cost of sex’ and is supported by theoretical and empirical evidence [23,25]. Parthenogenesis may also be less physically costly than sexual reproduction, as it does not require the location and selection of appropriate mates [26]. Finally, parthenogenetic reproduction may be particularly beneficial where environmental conditions are stable, as it allows beneficial allelic combinations to be retained across generations without meiotic recombination [27]. However, parthenogenesis also has several major limitations as a sustainable reproductive strategy. Namely, asexual reproduction results in a marked loss of genetic variation [28,29], impacting long-term population persistence, particularly in spatio-temporally heterogeneous environments [30]. Additionally, there is some evidence that offspring produced by facultative parthenogenesis suffer from reduced fitness in the form of both growth rates and survival, and elevated chances of exhibiting recessive genetic disorders (e.g. [31]). In species capable of facultative parthenogenesis for which sexual reproduction is nonetheless the norm, the net fitness benefit of asexual reproduction will generally be lower than that of sexual reproduction, but greater than zero where viable offspring can be produced (figure 1c).

Despite these apparent limitations, facultative parthenogenesis has been reported in at least 12 chondrichthyan species (table 1) representing 4 of the 14 extant orders [19,28,32–34,36,41–45]. Typically, parthenogenesis is first considered upon reports of females housed in isolation from male conspecifics successfully birthing offspring or depositing eggcases, and genetic analyses are subsequently used to rule out sperm storage [19,44]. This is important as female sperm storage in chondrichthyans is abundant and can result in viable offspring being produced at least 3.5 years after the most recent copulation event [46,47]. As all known records (bar one, see [43] or [42]) of parthenogenesis in chondrichthyans are from individuals housed in aquaria, biometric and physiological monitoring of both the mother and offspring is generally possible, providing important insights into offspring survival and the conditions under which facultative parthenogenesis might occur [31,36,38]. Alternatively, analyses of DNA microsatellite data obtained from wild *Pristis pectinata* individuals have shown the viability and persistence of juvenile parthenogens in a natural setting [43].

**Table 1.** Chondrichthyan species in which parthenogenesis has been reported. Note that in all but one case (*Rhynchobatus australiae*), parthenogenesis has been confirmed genetically.

order	species	reference(s)
Carcharhiniformes	*Carcharhinus limbatus*	[32]
	*Cephaloscyllium ventriosum*	[33]
	*Mustelus mustelus*	[19]
	*Sphyrna tiburo*	[28]
	*Triaenodon obesus*	[34]
Orectolobiformes	*Stegostoma tigrinum*	[31,35–38]
	*Chiloscyllium plagiosum*	[39–41]
Rhinopristiformes	*Pristis pectinata*	[42,43]
	*Rhynchobatus australiae*	[44]
	*Rhina ancylostoma*	[44]
Myliobatiformes	*Aetobatus narinari*	[45]
	*Himantura uarnak*	[44]

Unfortunately, few studies provide empirical data regarding the potential fitness consequences of facultative parthenogenesis in Chondrichthyes. Several studies have speculated that parthenogenesis may be an adaptive reproductive strategy to compensate for the absence of males [19,28,36], which would otherwise result in a complete loss of fitness once any stored sperm is used or becomes unviable (figure 1d). Indeed, most records of parthenogenesis in chondrichthyans come from isolated females held in captivity in the complete absence of males [19,28,32,34,36,39,41]. Moreover, in some cases, parthenogenesis seems to have been triggered rapidly following the removal of male conspecifics [36]. Similar observations have been made from other vertebrates [21]. The viability of elasmobranch parthenogens in both captive [39] and wild settings [42,43] provides further support for the idea that this alternative reproductive strategy may be adaptive. Recurrent and even second-generation parthenogenesis has been documented in some species [38,40]. Finally, parthenogenesis has been recorded in the presence of male conspecifics and following artificial insemination, indicating that the adaptive role of parthenogenesis may not relate purely to the male abundance, but also to perceived male quality [35,37,41]. In this context, male quality refers to the evolutionary fitness or genetic quality of males as perceived by females, which directly impacts the fitness of offspring produced by reproduction with said male [48]. The paucity of direct studies of sexual selection in chondrichthyans means that we have little understanding of mate choice dynamics in this clade, although both male and female mate choice have been suggested previously [49–51].

However, support for parthenogenesis as an adaptive reproductive strategy in Chondrichthyes is not universal. Most significantly, Adams *et al*. [31] reported various, substantial fitness costs of parthenogenesis. Relative to sexually produced counterparts, parthenogenetically produced zebra sharks (*Stegostoma tigrinum*) hatched at a smaller size and exhibited consistently lower growth rates [31]. In addition, parthenotes exhibited an array of unusual behaviours and apparent morphological abnormalities, with all individuals perishing prior to sexual maturity [31]. This observation provides support for the hypothesis that isolated cases of parthenogenesis in species that typically reproduce sexually result from errors during meiotic divisions rather than an adaptive strategy (see [21] and [52]). However, as referenced previously, there are multiple documented cases of viable parthenogens across different species (e.g. [33,39,42,43]), indicating that these fitness costs are not an intrinsic consequence of parthenogenesis in chondrichthyans.

On the basis of the available evidence, it appears likely that facultative parthenogenesis does represent an adaptive reproductive strategy in chondrichthyans, rather than a reproductive aberration resulting from imperfect meiosis. However, the exact conditions under which parthenogenesis should be favoured are uncertain; minimally, the local abundance of males over the reproductive lifespan of a female should be important. Even if parthenogenesis incurs substantial net fitness costs in chondrichthyans, this would be preferable to sexual reproduction if the absence of males extends beyond the time frame within which sperm storage represents a viable alternative, and if there are no prospects of sexual reproduction in the near future. However, external environmental factors are also probably important in determining the viability of parthenogenesis at any point in time. As mentioned previously, asexual reproduction is generally more advantageous when environmental conditions are stable [30]. Additionally, where facultative parthenogenesis impacts size and growth rates (e.g. [31]), local community composition and predation rates may exert influence over the net fitness consequences of parthenogenesis. Finally, where parthenogenesis occurs in the presence of conspecific males (presumably via female choice, see [37] or artificially introduced semen [35,41], the perceived genetic quality of available males could also influence the adaptive benefit of parthenogenesis. While not altering the net fitness consequences of parthenogenesis, the prevalence of ‘low quality’ males in the local environment would reduce the fitness of offspring produced by sexual reproduction, which could in turn ‘tilt the balance’ in favour of parthenogenesis.

## 3. Multiple embryos per eggcase

Unlike facultative parthenogenesis, cases of monozygotic twinning and polyembryony in chondrichthyans are typically regarded as aberrations resulting in reduced offspring fitness or increased mortality due to oxidative stress [12]. As such, monozygotic twinning appears to be extremely rare and has only been documented in a handful of cases [11,12,14,53]. However, a related phenomenon, in which multiple embryos developing from separate yolk sacs within a single eggcase (herein referred to as MEPE), appears to represent an adaptive reproductive strategy in at least two chondrichthyan species [20,54,55]. The term MEPE is chosen here instead of polyembryony as the genetic identity of offspring produced in this manner (i.e. whether they share paternity or not) remains unknown. In most oviparous elasmobranchs, it is assumed that a single embryo typically develops within each eggcase and thus that the number of eggcases deposited per reproductive cycle is directly proportional to fecundity and consequently to fitness. However, two species of skate (*Beringraja binoculata* and *Beringraja pulchra*) regularly produce eggcases containing two or more (up to eight) embryos developing from separate yolk sacs [54,55]. While the number of embryos (and rates of mortality) is highly variable, the optimal number of embryos per eggcase in *B. binoculata* is thought to be two in captivity [20] and three to four in the wild [56], indicating at least a potential doubling of fecundity relative to species in which only a single embryo develops per eggcase. Subsequently, a single further case of MEPE was described in the small-spotted catshark (*Scyliorhinus canicula*), with two embryos attached to different yolk sacs surviving to hatch and beyond [11]. Hook *et al*. [11] reported a potential case of MEPE in a fourth species (*Raja undulata*); however, it was not possible to conclusively determine whether embryos shared a yolk sac or not, and hence, monozygotic twinning (rather than MEPE) cannot be ruled out. Besides these four species, the extent to which MEPE occurs across chondrichthyan diversity remains unexplored.

While monozygotic twinning in elasmobranchs appears to commonly result in the death of embryos [11,12,14], this does not seem to be the case with MEPE [11,20,56]. Indeed, in the case of *B. binoculata*, eggcases containing up to five embryos have hatched without issue and eggcases containing a single embryo exhibited higher mortality than those with multiple [11]. Singular cases of MEPE (e.g. *S. canicula*) could result from mistiming between ovulation and encapsulation, and therefore represent reproductive aberrations. However, the frequency with which MEPE occurs in *B. binoculata* and *B. pulchra* suggests that it is a viable reproductive strategy in some chondrichthyans and may have adaptive value. Indeed, MEPE has been shown to be heritable in some other vertebrate lineages, with several candidate genes identified that are associated with reproductive traits across vertebrate diversity [57]. One caveat with this adaptive interpretation is that eggcases containing multiple embryos tend to be larger (requiring greater material investment) than those containing single embryos [11,20,54–56] and that nothing is known about if or how fecundity varies over entire reproductive cycles between species utilizing MEPE and those producing only single-yolked eggcases. However, in all reported cases of MEPE, eggcases are still smaller than the sum total of eggcases if each embryo was to have developed separately, and there is no evidence to suggest that MEPE influences the size or growth rates of embryos [11,20]. On this basis, MEPE could facilitate an increase in fecundity by reducing the total maternal investment required per offspring (e.g. the resources required to produce multiple eggcases) and increasing the amount of space in the body cavity for additional embryos. This latter point is important, given that female body size in elasmobranchs is thought to be under strong selection due to the need to devote space in the body cavity to reproduction [58]. Even if space in the female body cavity is not the limiting factor on fecundity, the reduction in eggcase size relative to the number of embryos produced would theoretically elevate fecundity at any given level of maternal investment. Consequently, MEPE represents one mechanism by which fecundity can be increased without requiring major evolutionary shifts in body size or maternal investment.

## 4. Open questions and future directions: population level effects

Population-level effects of reproductive variation in sharks and rays have been speculated, but at present, there is little evidence to support any such relationships [18]. The question of whether phenomena such as MEPE or facultative parthenogenesis could impact population status/vulnerability in this clade remains unanswered. Theoretically, increases in fecundity conveyed by either of these traits and the potential effects of parthenogenesis on offspring genetic diversity [22] could have notable consequences for population persistence. Fecundity is thought to be a key factor underlying the resilience of declining populations in the face of anthropogenic overexploitation, a threat facing many chondrichthyan populations [59]. However, despite the theoretical predictions (figure 1; [20]), we do not have enough data regarding the lifetime reproductive outputs of different chondrichthyan taxa to robustly determine whether parthenogenesis or MEPE influence fecundity across temporal scales greater than single reproductive events. Given the wealth of information that has been gathered about chondrichthyan reproductive biology from aquaria [44], rigorous and comprehensive records of fecundity, growth rates, reproductive behaviours and other data may help reduce such uncertainty in the future. In light of the often rapid transitions from sexual to parthenogenetic reproduction, I contend that comprehensive data collection of this type should be applied to all individuals held in aquaria, regardless of the perceived utility of the data at the time.

The effects of parthenogenesis and MEPE on genetic diversity could also feasibly have population-level effects. Almost all obligate parthenogenetic vertebrates have originated recently, implying that such lineages have relatively short lifespans and quickly succumb to fitness costs associated with elevated homozygosity [22]. Some evidence for these costs is evident in chondrichthyans [35], although it is unknown how widespread they might be. Alternatively, Dudgeon *et al*. [36] speculated that facultative parthenogenesis could facilitate population persistence during bottleneck events. However, there is no empirical evidence to support this hypothesis in Chondrichthyes, and indeed it may be impossible to test. Ultimately, the relevance of facultative parthenogenesis to population persistence in chondrichthyans is dependent on how frequently this reproductive strategy is utilized. Incidentally, while the effects of parthenogenesis on genetic variation are fairly well understood, the same is not true of MEPE. Only two studies have investigated genetic diversity of offspring produced via MEPE [11,56]. In the case of *S. canicula*, results were consistent with heteropaternal superfecundation, implying that MEPE may have no effect on genetic diversity whatsoever [11]. Consequently, genetic investigations of MEPE across larger sample sizes and other taxa (particularly those in which it appears most abundant) are warranted and may shed further light onto long-term implications of MEPE as an adaptive strategy.

## 5. Open questions and future directions: cues and triggers

Assuming facultative parthenogenesis is adaptive in Chondrichthyes, females may use cues to actively switch between sexual and asexual reproduction [36]. As the theoretical fitness consequences of parthenogenesis depend largely on the abundance of males over the reproductive lifespan of females (figure 1), it is plausible that such cues could represent a direct or indirect proxy for local male abundance. In line with this, Dudgeon *et al*. [36] suggested that the removal of male conspecifics from aquaria housing may trigger parthenogenesis. However, no explanation was given as to what the underlying cue used to detect the presence or absence of mates could be. In the case of aquaria, visual cues could suffice; however, this is clearly not realistic in natural settings, where distribution and habitat usage are far less constrained. In some other taxa, chemical cues associated with population size (which can be reinforced across generations by positive feedback and transgenerational maternal effects) induce switches between asexual and sexual reproduction [60,61], but such cues have yet to be described in any chondrichthyan taxa. Alternatively, cues could be related to the passing of some critical threshold of time since the last encounter with male conspecifics, or after stored sperm has been fully utilized. Given the prevalence of sexual segregation in elasmobranchs [62], it is also plausible that environmental cues relating to temperature, photoperiod or prey abundance could act as indirect proxies for local mate abundance. Similar environmental cues, which are not always linked to mate abundance, have been found to induce switches between sexual and asexual reproduction in other taxa [61,63]. If cues do not relate either directly or indirectly to mate presence/absence (as must be the case where parthenogenesis has occurred in the presence of male conspecifics, see [37]), cues could instead relate to mate quality. While experimental studies have recovered evidence for such cues in other taxa, it must be stressed that there is absolutely no evidence whatsoever for any of these mechanisms in chondrichthyan taxa. It is also possible that multiple cues/triggers may act concurrently, or over different timescales. To better understand potential cues underlying switches between sexual and asexual reproduction in this lineage, rigorous and detailed record keeping of captive populations (as outlined previously) must be prioritized, including comprehensive analyses of water chemistry. This would generate a larger dataset of circumstantial evidence indicating conditions that favour the induction of parthenogenesis.

In the case of MEPE, cues are not so relevant, at least in taxa for which MEPE appears to be the norm (*Beringraja* spp.). However, potential cues of MEPE are highly relevant in the case of *S. canicula*, in which MEPE has been recorded once [11] and so occurs at some unknown frequency within populations. Even in the case of *Beringraja* spp., it remains unknown what governs the high degree of variance in the number of embryos deposited in each eggcase [20]. In domesticated birds, ‘double-yolked’ eggs are a rare but well-documented phenomenon, commonly associated with the provisioning of excessive feed or ‘over-nutrition’ [57]. It is plausible that rare cases of MEPE in species such as *S. canicula* result from abnormally high foraging success and that variance in the number of embryos deposited per eggcase in *Beringraja* spp. results from variance in body condition. However, there are currently no data to support or refute this hypothesis. Given that MEPE involves not only the deposition of multiple embryos within a single eggcase but also an increase in eggcase size, further study is warranted to understand the physiological and regulatory basis of the relationship between these traits.

## 6. Open questions and future directions: taxon coverage

A final, equally important open question regarding these alternative reproductive strategies is the extent to which they are present across chondrichthyan diversity. Even if facultative parthenogenesis or MEPE are adaptive, we have little understanding of how prevalent these strategies may be in natural settings and in how many species they are used. Facultative parthenogenesis has been recorded in only 12 species (table 1), representing less than 1% of extant chondrichthyan diversity. It has been suggested that parthenogenetic reproduction may be far more prevalent than currently appreciated, on the basis that detailed monitoring and genetic analyses of offspring typically only occur following observations of reproduction in aquaria in the absence of males [44]. MEPE has only been confirmed in three species and appears to be far rarer than facultative parthenogenesis. While facultative parthenogenesis is known from many vertebrate lineages [22], the presence of multiple separate embryos within a single egg structure is an uncommon occurrence across vertebrate diversity and commonly results in embryo death [57,64–66]. It could be argued that if these reproductive strategies are isolated to a minute fraction of chondrichthyan taxa, they are of little significance to the broader fields of chondrichthyan reproductive or evolutionary biology. However, we simply do not know whether this is the case. Both strategies are present in sharks and rays (table 1; [11,20]), meaning there is little reason to suggest they should be restricted to certain orders or families. To overcome this uncertainty, I suggest that the molecular, DNA microsatellite-based approach outlined in Fields *et al*. [43] should be applied to as many species as possible in natural settings, even where there is no *a priori* reason to suggest parthenogenetic reproduction. In the case of MEPE, detailed analyses of eggcases recovered from fisheries and produced in captive settings are needed, to determine both the range of species in which MEPE might occur and the frequency with which MEPE yields viable offspring.

## 7. Conclusions

Facultative parthenogenesis and MEPE have been documented in multiple vertebrate taxa and theoretically result in exponential increases in fecundity. However, both traits additionally have a number of costs that leave their net fitness consequences uncertain, particularly in chondrichthyan taxa for which we have comparatively little understanding of reproductive biology. On the basis of the existing evidence, it seems likely that both traits are adaptive, at least in some taxa and ecological contexts. While we cannot rule out either trait being abundant across chondrichthyan diversity, at present reports are restricted to a small number of species. Moreover, we entirely lack the necessary quantitative data to infer the consequences of facultative parthenogenesis or MEPE for lifetime fecundity. This uncertainty underscores a broader lack of information regarding reproductive variation in Chondrichthyes and across vertebrate diversity.

To overcome this uncertainty, it will be necessary to better quantify the costs and benefits of diverse reproductive strategies across a range of spatio-temporal contexts and across a range of taxa. In the case of Chondrichthyes, a combination of improved record-keeping of populations housed in aquaria, combined with application of emerging genetic and genomic technologies, should be a key focus of future research, in order to develop a better understanding of the ecological contexts and diversity of species in which these traits are observed. The conservation status of many chondrichthyan populations is dire, and further study of reproductive traits that theoretically increase fecundity is needed to gain insight into the effects of life history and reproductive variation on vulnerability.

## Data Availability

This article has no additional data.

## References

[1] Wourms JP. 1977 Reproduction and development in chondrichthyan fishes. Am. Zool. **17**, 379–410. (10.1093/icb/17.2.379)

[2] Angelini F, Ghiara G. 1984 Reproductive modes and strategies in vertebrate evolution. Ital. J. Zool. **51**, 121–203.

[3] Blackburn DG. 1992 Convergent evolution of viviparity, matrotrophy, and specializations for fetal nutrition in reptiles and other vertebrates. Am. Zool. **32**, 313–321. (10.1093/icb/32.2.313)

[4] Blackburn DG. 1999 Viviparity and oviparity: evolution and reproductive strategies. Encycl. Reprod. **4**, 994–1003.

[5] Lodé T. 2012 Oviparity or viviparity? That is the question…. Reprod. Biol. **12**, 259–264. (10.1016/j.repbio.2012.09.001) 23153695

[6] Katona G, Szabó F, Végvári Z, Székely T Jr, Liker A, Freckleton RP, Vági B, Székely T. 2023 Evolution of reproductive modes in sharks and rays. J. Evol. Biol. **36**, 1630–1640. (10.1111/jeb.14231) 37885147

[7] Conrath CL, Musick JA. 2012 Reproductive biology of elasmobranchs. In Biology of sharks and their relatives (eds JC Carrier, CA Simpfendorfe, MR Heithaus, KE Yopak), pp. 291–311, 2nd edn. Boca Raton, FL: CRC Press.

[8] Gayford JH, Jambura PL. 2025 Drivers of diversification in sharks and rays (Chondrichthyes: Elasmobranchii). Front. Ecol. Evol. **12**, fevo.2024.1530326. (10.3389/fevo.2024.1530326) 40027935 PMC7617448

[9] Mull CG, Pennell MW, Yopak KE, Dulvy NK. 2024 Maternal investment evolves with larger body size and higher diversification rate in sharks and rays. Curr. Biol. **34**, 2773–2781.(10.1016/j.cub.2024.05.019) 38843829

[10] Blackburn DG, Hughes DF. 2024 Phylogenetic analysis of viviparity, matrotrophy, and other reproductive patterns in chondrichthyan fishes. Biol. Rev. Camb. Philos. Soc. **99**, 1314–1356. (10.1111/brv.13070) 38562006

[11] Hook SA, Musa SM, Ripley DM, Hibbitt JD, Grunow B, Moritz T, Shiels HA. 2019 Twins! Microsatellite analysis of two embryos within one egg case in oviparous elasmobranchs. PLoS One **14**, e0224397. (10.1371/journal.pone.0224397) 31790403 PMC6886835

[12] Luer CA, Wyffels JT. 2022 Selected topics in the developmental biology of chondrichthyan fishes. In Biology of sharks and their relatives (eds JC Carrier, CA Simpfendorfer, MR Heithaus, KE Yopak), pp. 251–288. Boca Raton, FL: CRC Press. (10.1201/9781003262190-9)

[13] Gilmore RG, Putz O, Dodrill JW. 2011 Oophagy, intrauterine cannibalism and reproductive strategy in lamnoid sharks. In Reproductive biology and phylogeny of chondrichthyes (ed. WC Hamlett), pp. 435–462. Boca Raton, FL: CRC Press.

[14] Gajić AA, de Loose E, Martin AG, Neuman E, Karalić E, Beširović H, Gayford JH. 2025 Two’s company: monozygotic twinning in the small-spotted catshark (Scyliorhinus canicula). J. Fish Biol. (10.1111/jfb.70049) PMC12360130 40195855

[15] Byrne RJ, Avise JC. 2012 Genetic mating system of the brown smoothhound shark (Mustelus henlei), including a literature review of multiple paternity in other elasmobranch species. Mar. Biol. **159**, 749–756. (10.1007/s00227-011-1851-z)

[16] Pratt HL, Tanaka SHO. 1994 Sperm storage in male elasmobranchs: a description and survey. J. Morphol. **219**, 297–308. (10.1002/jmor.1052190309) 8169955

[17] Dutilloy A, Dunn MR. 2020 Observations of sperm storage in some deep-sea elasmobranchs. Deep Sea Res. Part I **166**, 103405. (10.1016/j.dsr.2020.103405)

[18] Gayford JH, Flores-Flores EM. 2024 No evidence for population-level benefits of polyandry in sharks and rays. PLoS One **19**, e0308141. (10.1371/journal.pone.0308141) 39231154 PMC11373851

[19] Esposito G *et al*. 2024 First report of recurrent parthenogenesis as an adaptive reproductive strategy in the endangered common smooth-hound shark Mustelus mustelus. Sci. Rep. **14**, 17171. (10.1038/s41598-024-67804-1) 39060341 PMC11282070

[20] Chiquillo KL, Ebert DA, Slager CJ, Crow KD. 2014 The secret of the mermaid’s purse: phylogenetic affinities within the Rajidae and the evolution of a novel reproductive strategy in skates. Mol. Phylogenetics Evol. **75**, 245–251. (10.1016/j.ympev.2014.01.012) PMC4036632 24486989

[21] Lampert KP. 2008 Facultative parthenogenesis in vertebrates: reproductive error or chance? Sex. Dev. **2**, 290–301. (10.1159/000195678) 19276631

[22] Fujita MK, Singhal S, Brunes TO, Maldonado JA. 2020 Evolutionary dynamics and consequences of parthenogenesis in vertebrates. Annu. Rev. Ecol. Evol. Syst. **51**, 191–214. (10.1146/annurev-ecolsys-011720-114900)

[23] Lehtonen J, Jennions MD, Kokko H. 2012 The many costs of sex. Trends Ecol. Evol. **27**, 172–178. (10.1016/j.tree.2011.09.016) 22019414

[24] Maynard-Smith J. 1978 The evolution of sex. vol. 4. Cambridge, UK: Cambridge University Press.

[25] Gibson AK, Delph LF, Lively CM. 2017 The two-fold cost of sex: experimental evidence from a natural system. Evol. Lett. **1**, 6–15. (10.1002/evl3.1) 30233811 PMC6089407

[26] Daly M. 1978 The cost of mating. Am. Nat. **112**, 771–774. (10.1086/283319)

[27] Agrawal AF. 2006 Evolution of sex: why do organisms shuffle their genotypes? Curr. Biol. **16**, R696–R704. (10.1016/j.cub.2006.07.063) 16950096

[28] Chapman DD, Shivji MS, Louis E, Sommer J, Fletcher H, Prodöhl PA. 2007 Virgin birth in a hammerhead shark. Biol. Lett. **3**, 425–427. (10.1098/rsbl.2007.0189) 17519185 PMC2390672

[29] Hedrick PW. 2007 Virgin birth, genetic variation and inbreeding. Biol. Lett. **3**, 715–716. (10.1098/rsbl.2007.0293) 17698447 PMC2391206

[30] Holmes CM, Nemenman I, Weissman DB. 2018 Increased adaptability to sudden environmental change can more than make up for the two-fold cost of males. Europhys. Lett **123**, 58001. (10.1209/0295-5075/123/58001)

[31] Adams L, Lyons K, Monday J, Larkin E, Wyffels J. 2023 Costs of parthenogenesis on growth and longevity in ex situ zebra sharks Stegostoma tigrinum. Endanger. Species Res. **50**, 81–91. (10.3354/esr01224)

[32] Chapman DD, Firchau B, Shivji MS. 2008 Parthenogenesis in a large‐bodied requiem shark, the blacktip Carcharhinus limbatus. J. Fish Biol. **73**, 1473–1477. (10.1111/j.1095-8649.2008.02018.x)

[33] Feldheim KA *et al*. 2017 Multiple births by a captive swellshark Cephaloscyllium ventriosum via facultative parthenogenesis. J. Fish Biol. **90**, 1047–1053. (10.1111/jfb.13202) 27861877

[34] Portnoy DS, Hollenbeck CM, Johnston JS, Casman HM, Gold JR. 2014 Parthenogenesis in a whitetip reef shark Triaenodon obesus involves a reduction in ploidy. J. Fish Biol. **85**, 502–508. (10.1111/jfb.12415) 24905881

[35] Adams L, Lyons K, Larkin E, Leier N, Monday J, Plante C, Dubach J, Wyffels J. 2022 Artificial insemination and parthenogenesis in the zebra shark Stegostoma tigrinum. Front. Mar. Sci. **9**, 886616. (10.3389/fmars.2022.886616)

[36] Dudgeon CL, Coulton L, Bone R, Ovenden JR, Thomas S. 2017 Switch from sexual to parthenogenetic reproduction in a zebra shark. Sci. Rep. **7**, 1–8. (10.1038/srep40537) 28091617 PMC5238396

[37] Feldheim KA, Dubach J, Watson L. 2023 Parthenogenesis in an elasmobranch in the presence of conspecific males. J. Fish Biol. **102**, 525–527. (10.1111/jfb.15268) 36369968

[38] Robinson DP, Baverstock W, Al‐Jaru A, Hyland K, Khazanehdari KA. 2011 Annually recurring parthenogenesis in a zebra shark Stegostoma fasciatum. J. Fish Biol. **79**, 1376–1382. (10.1111/j.1095-8649.2011.03110.x) 22026614

[39] Feldheim KA, Chapman DD, Sweet D, Fitzpatrick S, Prodohl PA, Shivji MS, Snowden B. 2010 Shark virgin birth produces multiple, viable offspring. J. Hered. **101**, 374–377. (10.1093/jhered/esp129) 20106913

[40] Straube N, Lampert KP, Geiger MF, Weiß JD, Kirchhauser JX. 2016 First record of second‐generation facultative parthenogenesis in a vertebrate species, the whitespotted bambooshark Chiloscyllium plagiosum. J. Fish Biol. **88**, 668–675. (10.1111/jfb.12862) 26727105

[41] Wyffels JT, Adams LM, Bulman F, Fustukjian A, Hyatt MW, Feldheim KA, Penfold LM. 2021 Artificial insemination and parthenogenesis in the whitespotted bamboo shark Chiloscyllium plagiosum. Sci. Rep. **11**, 9966. (10.1038/s41598-021-88568-y) 33980873 PMC8116330

[42] Feldheim KA, Fields AT, Chapman DD, Scharer RM, Poulakis GR. 2017 Insights into reproduction and behavior of the smalltooth sawfish Pristis pectinata. Endanger. Species Res. **34**, 463–471. (10.3354/esr00868)

[43] Fields AT, Feldheim KA, Poulakis GR, Chapman DD. 2015 Facultative parthenogenesis in a critically endangered wild vertebrate. Curr. Biol. **25**, R446–R447. (10.1016/j.cub.2015.04.018) 26035783

[44] Feldheim KA, Wyffels JT, Lyons K. 2022 The role of aquaria in the advancement of elasmobranch reproductive biology. Front. Mar. Sci. **9**, 963542. (10.3389/fmars.2022.963542)

[45] Harmon TS, Kamerman TY, Corwin AL, Sellas AB. 2016 Consecutive parthenogenetic births in a spotted eagle ray Aetobatus narinari. J. Fish Biol. **88**, 741–745. (10.1111/jfb.12819) 26563982

[46] Bernal MA, Sinai NL, Rocha C, Gaither MR, Dunker F, Rocha LA. 2015 Long‐term sperm storage in the brownbanded bamboo shark Chiloscyllium punctatum. J. Fish Biol. **86**, 1171–1176. (10.1111/jfb.12606) 25545440

[47] Pratt HL. 1993 The storage of spermatozoa in the oviducal glands of western North Atlantic sharks. In The reproduction and development of sharks, skates, rays and ratfishes developments in environmental biology of fishes (eds LS Demski, JP Wourms), pp. 139–149. Dodrecht, The Netherlands: Springer. (10.1007/978-94-017-3450-9_12)

[48] Burley N. 1977 Parental investment, mate choice, and mate quality. Proc. Natl Acad. Sci. USA **74**, 3476–3479. (10.1073/pnas.74.8.3476) 269407 PMC431613

[49] Lyons K, Kacev D, Mull CG. 2021 An inconvenient tooth: evaluating female choice in multiple paternity using an evolutionarily and ecologically important vertebrate clade. Mol. Ecol. **30**, 1574–1593. (10.1111/mec.15844) 33586211 PMC8251896

[50] Ritter EK, Amin RW. 2019 Mating scars among sharks: evidence of coercive mating? Acta Ethologica **22**, 9–16. (10.1007/s10211-018-0301-z)

[51] Whitehead DA, Gayford JH, Hoyos EM, Shorter NM, Galván-Magaña F, Ketchum JT. 2022 First description of a sex segregated aggregation of silky sharks (Carcharhinus falciformis) and the frequency and distribution of mating wounds off the tip of the Baja California Peninsula. Environ. Biol. Fishes **105**, 953–960. (10.1007/s10641-022-01297-7)

[52] Schwander T, Vuilleumier S, Dubman J, Crespi BJ. 2010 Positive feedback in the transition from sexual reproduction to parthenogenesis. Proc. R. Soc. B **277**, 1435–1442. (10.1098/rspb.2009.2113) PMC2871946 20071382

[53] Ivanoff R, Vooren CM. 2015 On growth and development of monozygotic twin embryos of the shortnose spurdog Squalus megalops (Macleay, 1881) (Elasmobranchii: Squalidae). Pan Am. J. Aquat. Sci **10**, 134–140.

[54] Ishiyama R. 1958 Observations on the egg-capsules of skates of the family Rajidae found in Japan and its adjacent waters. Bull. Mus. Comp. Zool. **118**, 1–24.

[55] Hitz CR. 1964 Observations on egg cases of the big skate (Raja binoculata Girard) found in Oregon coastal waters. J. Fish. Board Can. **21**, 851–854.

[56] Jang JJJ. 2019 Reproductive strategies of the big skate (Beringraja binoculata) with evidence of multiple paternity. Master’s thesis, California State University, Monterey Bay, CA.

[57] Salamon A. 2020 Factors affecting the production of double-yolked eggs. World’s Poult. Sci. J. **76**, 815–826. (10.1080/00439339.2020.1830011)

[58] Gayford JH, Sternes PC. 2024 The origins and drivers of sexual size dimorphism in sharks. Ecol. Evol. **14**, e11163. (10.1002/ece3.11163) 38500855 PMC10944705

[59] Dulvy NK *et al*. 2021 Overfishing drives over one-third of all sharks and rays toward a global extinction crisis. Curr. Biol. **31**, 4773–4787.(10.1016/j.cub.2021.08.062) 34492229

[60] Gerritsen J. 1980 Sex and parthenogenesis in sparse populations. Am. Nat. **115**, 718–742. (10.1086/283594)

[61] Zadereev YS. 2003 Maternal effects, conspecific chemical cues, and switching from parthenogenesis to gametogenesis in the cladoceran Moina macrocopa. Aquat. Ecol. **37**, 251–255.

[62] Wearmouth VJ, Sims DW. 2010 Sexual segregation in elasmobranchs. Biol. Mar. Mediterr. **17**, 236.

[63] Markovitch S. 1924 The migration of the Aphididae and the appearance of sexual forms as affected by the relative length of daily exposure. J. Agric. Res. **27**, 513–522.

[64] Jeffrey FP, Fox TW, Smyth JJ. 1953 Observations on double-yolked eggs from the domestic fowl. J. Hered. **44**, 213–216.

[65] Lee KH, Fechheimer NS, Abplanalp H. 1990 Euploid chicken embryos from eggs containing one, two or several yolks. Reproduction **89**, 85–90. (10.1530/jrf.0.0890085) 2374135

[66] Marion KR. 1980 One-egg twins in a snake, Elaphe guttata guttata. Trans. Kans. Acad. Sci. **83**, 98–100. (10.2307/3627722)

